# Numerical simulation of novel concept 4D cardiac microtomography for small rodents based on all-optical Thomson scattering X-ray sources

**DOI:** 10.1038/s41598-019-44779-y

**Published:** 2019-06-11

**Authors:** Daniele Panetta, Luca Labate, Lucia Billeci, Nicole Di Lascio, Giuseppina Esposito, Francesco Faita, Giovanni Mettivier, Daniele Palla, Luciano Pandola, Pietro Pisciotta, Giorgio Russo, Antonio Sarno, Paolo Tomassini, Piero A. Salvadori, Leonida A. Gizzi, Paolo Russo

**Affiliations:** 1 Consiglio Nazionale delle Ricerche, Istituto di Fisiologia Clinica, 56124 Pisa, Italy; 2 Consiglio Nazionale delle Ricerche, Istituto Nazionale di Ottica, 56124 Pisa, Italy; 3 INFN, Sezione di Pisa, 56127 Pisa, Italy; 4 Università di Napoli Federico II, Dipartimento di Fisica “Ettore Pancini”, 80126 Napoli, Italy; 5 INFN, Laboratori Nazionali del Sud, 95123 Catania, Italy; 6 Consiglio Nazionale delle Ricerche, Istituto di Bioimmagini e Fisiologia Molecolare, 90015 Cefalù, Italy

**Keywords:** Ultrafast lasers, Biological physics

## Abstract

Accurate dynamic three-dimensional (4D) imaging of the heart of small rodents is required for the preclinical study of cardiac biomechanics and their modification under pathological conditions, but technological challenges are met in laboratory practice due to the very small size and high pulse rate of the heart of mice and rats as compared to humans. In 4D X-ray microtomography (4D *μ*CT), the achievable spatio-temporal resolution is hampered by limitations in conventional X-ray sources and detectors. Here, we propose a proof-of-principle 4D *μ*CT platform, exploiting the unique spatial and temporal features of novel concept, all-optical X-ray sources based on Thomson scattering (TS). The main spatial and spectral properties of the photon source are investigated using a TS simulation code. The entire data acquisition workflow has been also simulated, using a novel 4D numerical phantom of a mouse chest with realistic intra- and inter-cycle motion. The image quality of a typical single 3D time frame has been studied using Monte Carlo simulations, taking into account the effects of the typical structure of the TS X-ray beam. Finally, we discuss the perspectives and shortcomings of the proposed platform.

## Introduction

Small rodents, like rats and mice, are the most useful and validated animal models for the study of cardiovascular diseases (CVD), and especially of myocardial ischemia (MI) and heart failure (HF)^1,2^. Surgical models of acute myocardial infarction (MI) and postischemic HF in rats are well validated and commonly employed^3,4^. Imaging technologies are of key importance for quantitative simultaneous evaluation of regional and global changes in myocardial contractility and perfusion; for this reason, great efforts have been made in the last two decades to implement scanners for the study of small laboratory animals, able to meet the high spatial and temporal resolution required by this type of animal model^5–7^. Limitations of current preclinical imaging scanners in terms of spatio-temporal resolution pose constraints on the accurate quantification of regional myocardial function/perfusion in small animal models of MI and HF. Due to the complex and fast three-dimensional motion of the heart, the identification of early regional dysfunction based on 3D strain and strain rate analysis would require real volumetric imaging at sub-millimeter spatial resolution and time-resolved imaging with more than 20–50 time frames per cardiac cycle^8^. High temporal resolution modalities such as micro magnetic resonance (micro-MRI) or micro-ultrasonography (micro-US) only allow multi-slice, non-isotropic (stacked 2D) imaging. On the other hand, micro-computed tomography (micro-CT) and micro-single photon emission tomography (micro-SPECT) with double ECG/respiratory gating do allow isotropic fully 3D imaging, even though cine-mode dynamic reconstructions are limited to 10 time frames per cardiac cycle or less^9,10^. In particular, dynamic micro-CT (4D *μ*CT) appears promising for morphofunctional imaging of the cardiac biomechanics, due to its high spatial resolution and good discrimination of myocardial walls, ventricular cavities and lung tissue upon use of suitable blood-pool contrast agents. Several 4D *μ*CT scanner designs have been validated by different investigators in the last decade, using either retrospective or prospective ECG gating. A full review of the existing systems and methods for 4D cardiac *μ*CT imaging is beyond the scope of this paper; for a list of the most relevant works, see the papers cited in Table 1 and references therein.

**Table 1:** Main parameters of X-ray sources suitable for application in 4D *μ*CT of the mouse heart.

X-ray source	Spot size	Pulse duration	Allows in-line phase contrast	Prosp. gating	Retrosp. gating	Notes	refs
Minifocus tubes	30–200 *μ*m	Continuous			•	Temporal resolution limited by the max. frame rate of X-ray detectors	^47, 66^
Medical tubes	300–800 *μ*m	>5 ms		•	•	Only with low magnification/Sub-optimal spectral quality for small animal imaging	^48, 67^
Carbon nanotube field emission X-ray tubes	>100 *μ*m	>100 *μ*s		•	•		^68^
Synchrotron hard X-ray beamlines (3rd generation)	Parallel beam	10–100 ps	•		•	4D CT *in-vivo* studies so far only applied to lung imaging/few facilities	^69, 70^
RF-based Thomson scattering	>40 *μ*m	10–20 ps	•		•	No cardiac 4D imaging studies reported so far	^13^
All-optical Thomson scattering (this work)	<10 *μ*m	<100 fs	•		•		—

Over the past few years, a novel kind of X-ray source, based upon the Thomson scattering (TS) of optical photons off relativistic electron bunches, has been intensively studied and developed worldwide^11,12^. For instance, a TS X-ray source based on conventional RF LINAC and miniaturized electron storage ring has been recently commercialized by Lyncean Technologies Inc. and used for dynamic phase contrast imaging of the lung of living mice^13^. From a practical point of view, the striking feature of a TS X-ray source lies in the possibility to produce radiation in the hard X-ray region using electrons with a much lower energy, usually in the range of a few tens up to a few hundreds of MeV, than that required in synchrotron or free-electron lasers (FEL) machines, due to the much favourable scaling of the emitted photon energy with the electron *γ* factor^11^. This, in turn, results in much smaller footprint and more affordable costs. The peak brightness of TS sources can exceed 10^20^ph/(s mm^2^ mrad^2^ 0.1%BW)^14,15^, only a couple of orders of magnitude smaller than that of 3rd generation light sources currently in operation (see for instance^16^). A TS source delivering photon beams in the hard X-ray region typically features an RF LINAC, accelerating electrons up to a few hundreds of MeV, and a pulsed laser system, providing optical photon beams with energy up to $\lesssim 1\,{\rm{J}}/{\rm{p}}{\rm{u}}{\rm{l}}{\rm{s}}{\rm{e}}$ and duration $\lesssim 1\,{\rm{ps}}$ (the “scattering beam”). The typical footprint of such machines is of the order of a few tens up to ~100 meters, which is typical of medium scale research infrastructures. On the other hand, laser-driven electron accelerators has been witnessing an impressive development over the past decade, making it an ideal candidate for replacing RF LINACs for applications in medicine and biology^17–19^, due to the reduced footprint and costs and the higher electron energy achievable using small scale devices. This kind of accelerator is based on the so-called Laser WakeField Acceleration (LWFA) process in a plasma (see^20,21^ for recent reviews); due to the very high field gradient which can be established in the accelerating plasma wave (up to 3 orders of magnitude higher than in a typical RF LINAC), electron bunches with energy up to the few GeVs level can be accelerated over a few up to a few tens of millimeters distances^22^, which is why LWFA sources have been dubbed “table-top” accelerators.

Based on this electron acceleration process, secondary X/*γ*-ray emission have been studied and successfully demonstrated^23^ over the past few years; several pilot application experiments have also been reported recently^24^, exploiting either the so-called betatron oscillation of the electrons into the accelerating plasma wave^25,26^ or the Thomson scattering mechanism^27,28^. Both these kinds of novel concept sources allow an extremely small spot size (down to $\lesssim 1\,\mu {\rm{m}}$) and an ultrashort pulse duration (down to the femtosecond range) to be achieved at the same time^11,29^. Static (3D) microtomographic applications have been demonstrated using betatron radiation from laser-driven electron accelerators^26,30^, even though the mean photon energy around 10–30 keV appears lower than optimal when working with living mice or rats, especially when using iodine-based contrast agents. While betatron sources can only deliver broadband photon spectra, Thomson scattering sources have the potential to produce narrow band X-ray beams, depending ultimately on the energy spread of the primary electron bunch. Since LWFA acceleration of electrons with low energy spread has already been demonstrated, all-optical TS sources offer a very promising route to get monochromatic radiation in the diagnostic energy range using table-top devices. Such “all-optical” X-ray sources are recognized as the most promising compact alternative to 3rd and 4th generation X-ray sources, providing ultrashort pulses and high peak brightness while keeping reasonable size and costs. The potential of these sources as a future replacement of current vacuum-tube technology in diagnostic and therapeutic regime is currently been investigated^18,19^.

In this paper, we investigate the use of an all-optical TS source for X-ray 4D *μ*CT. Our analysis will make it clear that such a kind of source meets all the requirements for such an application, namely: (a) the possibility to image out a sample of $\sim 2\times 5\,{{\rm{c}}{\rm{m}}}^{2}$ typical size, with a voxel size down to $\sim 100\,\mu {\rm{m}}$; (b) the possibility of getting a radiography with a single X-ray pulse (i.e., laser shot), which translates into a number of photons $\sim {10}^{3}/\mathrm{px}$, on a typical detector with ~100–200 *μ*m pixel size; (c) an X-ray pulse duration $\lesssim 100\,\mu {\rm{s}}$ (this point will be better deepened later); (d) an energy spectrum suitable to small animal imaging using iodine-based contrast agents (i.e., with a major part of the emission at $\gtrsim 33\,{\rm{keV}}$ energy, corresponding to the iodine *K*-edge); a repetition rate $\gtrsim 10\,{\rm{Hz}}$, so as to keep the overall *μ*CT duration limited to few tens of minutes. As it will be shown later, a laser-driven TS source also features a high spatial coherence, due to its source size being in the order of few micrometers, which makes it very attractive for phase contrast imaging. In order to demonstrate all these features, we rely on start-to-end simulations to get insights of issues such as pulse duration, photon statistics and energy spectrum using a realistic kinetic behaviour of cardiac wall implemented in a numerical 4D mouse chest phantom, as well as radiation dose and total duration of the experiment involving the living animal. First, a general overview of the envisioned TS 4D *μ*CT device will be given, including the results of a simulation of the spectral and spatial features of an all-optical X-ray source. Afterwards, the simulation results of a full retrospectively gated acquisition following the foreseen data acquisition scheme is presented. An account is also made of the expected final image quality and of the dosimetric issues, as resulting from Monte Carlo simulations using the actual photon distribution of a TS source. The main pro’s and con’s of our approach are then discussed.

## Design of the Laser-Based 4D *μ*CT Scanner Prototype

### Prototype layout

**Figure 1:**
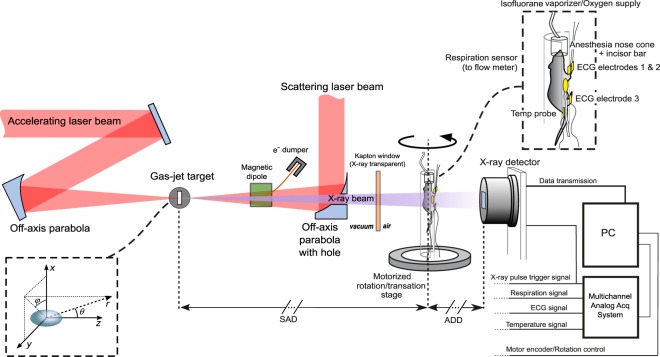
Conceptual scheme of the laser-based 4D *μ*CT scanner prototype for cardiac imaging of small rodents.

A conceptual layout of the proposed 4D *μ*CT scanner based on an all-optical TS source is shown in Fig. 1. Due to the inability (at least using simple setups) to put the TS source on a rotating gantry, the animal is placed on a cradle rotating around a vertical axis. The animal support is equipped with a physiological monitoring system (ECG, respiration, temperature), and a nose cone for gas anesthesia. The source-to-axis distance (SAD) and axis-to-detector distance (ADD) are kept flexible in our design; however, a sufficiently long ADD would enable the interesting spatial coherence properties of a TS source to be exploited for phase contrast imaging, although this issue will not be discussed here. All the physiological signals are acquired in a common time reference along with the laser pulse trigger and the image sequence grabbed by the X-ray detector. A/D sampling rate >2 kHz will be used for physiological signals, in order to ensure off-line identification of the R peaks of the ECG with sub-ms temporal precision (see Supplementary Materials). A conceptual sketch of the laser-driven X-ray source based on Thomson scattering is shown on the left side of Fig. 1. In detail, an ultrashort laser pulse (the “driving” pulse) is focused onto a gas-jet target, under vacuum, at a relativistic intensity^28^. The LWFA accelerated electron beam is set to collide with a second (“scattering”) laser beam just after having been accelerated (typically, for a well matched LWFA stage, at the exit of the gas-jet target). We defer until the next subsection a deeper presentation of the physical parameters of this source. We just observe here that, although requiring a complex setup (in order, for instance, to spatially and temporally overlap the accelerated electron bunch and the scattering laser pulse), the experimental apparatus envisioned here has been already demonstrated to be accessible, even on a routine basis, in a small scale laboratory (see for instance^31^ and refs therein).

The temporal rebinning scheme for tomographic acquisitions is fully described in the Supplementary Materials. Due to the extremely short duration of each pulse (~10^13^ times shorter than typical R-R interval in small animals), the likelihood that two different pulses will overlap in the same time bin of the cardiac cycle is negligible; hence, the maximum number of non-overlapping time bins will only depend on the total number of pulses acquired (i.e., constrained by the total absorbed dose and the total duration of the experiment). We believe that this extremely short pulse duration will open new possibilities in the field of cardiac imaging of small rodents, overcoming limitations of the current 4D *μ*CT systems with typical reconstruction windows of 5–10 ms per cardiac phase due to the constraints on pulse duration of conventional X-ray tubes and temporal resolution of X-ray detectors. Cooled scientific grade CCD or CMOS optically coupled or directly bonded to scintillators/phosphor screens appear the most adequate detection systems for the proposed application^26^.

### The Thomson Scattering Source

In this subsection, we will briefly discuss the main features of the all-optical Thomson scattering source envisioned for the advanced 4D *μ*CT scanner and we provide some relevant figures of the photon beam. As a preliminary observation, we stress that the main figures considered here for both the electron bunch (e.g., total charge per bunch, energy spectrum and divergence/emittance) and the scattering laser beam (e.g., pulse energy, duration and focal spot size) have been demonstrated to be achievable with existing (although state-of-the-art) laser systems. In particular, according to the current, consolidated literature on experimental achievements in the field of LWFA, the usage of a 100 TW class laser system could be suitable for the application discussed in this paper. As anticipated above, a 10Hz rep rate can be safely considered a standard value for such a class of lasers. The Thomson scattering source was modelled using the TSST code^32^, which provides the number of photons emitted in selected energy and solid angle bins over the desired ranges, once the parameters of the electron bunch (energy spectrum, spatial distribution and divergence, bunch charge), of the scattering laser pulse (central wavelength, duration and energy) and the geometry of the interaction are known. Details on the simulation code will be provided in the Methods.

The parameters of the LWFA bunch and the scattering laser beam used as input of the TSST simulation for the application discussed here are shown in Table 2 (left column). The scattering laser pulse, although picked off from the same laser chain as the main one, is stretched up to 500 fs duration^33^. We notice here that we took into account a “not so small” energy spread, which ultimately affects the spectral width of the X-ray photon beam^29^. Our choice for the energy spread is motivated by the ambition to keep the experimental setup of the X-ray source as simple as possible. Advanced schemes have been recently reported which allow remarkably lower energy spreads to be attained (such as, for instance, the usage of colliding pulses^34^ or the so-called ReMPI scheme^35^). However, these schemes either result, at the moment, in a rather low bunch charge (which is an essential parameter for the final photon flux^36^) or require a complex setup. Generally speaking, a trade-off does in fact exist between the possibility to get a monochromatic photon beam and the number of photons per laser shot. Here we require that a single image (that is, an image with an average number of photons/pixel as the one shown in the right column in Table 2) might be obtained using a single laser pulse. To this purpose, an electron bunch charge of $\sim 100\,{\rm{pC}}$ must be accelerated on each shot. Provided that the required energy spread is not too small (~15–20% rms), as it is in our case, such charge figures have been being reported in the literature over the past few years (see for instance^37^ or^38^). We also report in the Supplementary Materials on Particle-In-Cell (PIC) simulations, performed using the FBPIC code^39^, showing a possible LWFA regime delivering such bunches. We point out that, although all-optical TS sources may potentially provide X-ray beams with smaller spectral width, the rather large width considered here is not a limiting factor for the application discussed in this work; this issue will be addressed below using Monte Carlo simulations.

**Table 2:** *Left column*: main parameters relevant for the operating regime of the X-ray source; the electron bunch and scattering laser pulse figures were used as input of the Thomson scattering simulation code TSST.

Thomson scattering X-ray source parameters		Imaging simulation parameters	
**Electron bunch**		SAD	1000 mm
Size	2 *μ*m × 2 *μ*m (tr), 3 *μ*m (long)	ADD	1500 mm
Divergence	10 mrad	Magnification	2.5
Bunch charge	100 pC	Detector size (transverse × axial)	50 × 25 mm^2^
Mean energy	$\sim 50\,{\rm{MeV}}$	Detector pixel size	250 *μ*m
Energy spread	$\lesssim \,\mathrm{20 \% }$ rms	X-ray pulse duration	0
**Scattering laser pulse**		Total n. of X-ray pulses (for the entire 4D reconstruction)	6000 to 24000
Duration	500 fs (chirped)	Unattenuated no. photons per 250 *μ*m pixel	5000
Energy	1J	Unattenuated no. photons per 50 *μ*m pixel	200
Central wavelength	800 nm	Size of the cubic voxel in tomographic reconstructions	100 *μ*m
Spot size	20 *μ*m		
*a*_0_	~0.24		
**X-ray source**			
Duration	$\sim 10\,{\rm{fs}}$		
Source transverse size	2 *μ*m × 2 *μ*m		
No. of photons within 10 mrad per laser shot	$\sim 9.8\times {10}^{7}$		

More parameters related to a possible LWFA regime able to provide the required electron bunch are given in the Supplementary Materials. *Right column*: parameters of the numerical simulation with the 4D mouse chest phantom (SAD: Source to axis distance; ADD: Axis to detector distance).

**Figure 2:**
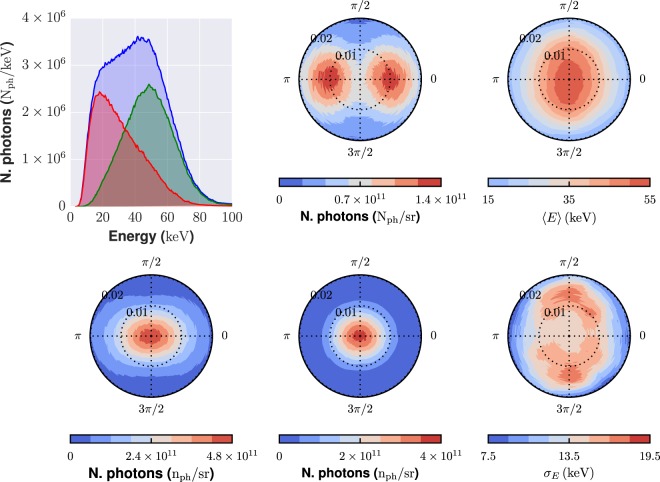
Spectral and angular distributions of the X-ray photons from the all-optical Thomson scattering source as retrieved using the TSST simulation code. The polar (*ϑ*) and azimuthal (*φ*) angles are defined in Fig. 1. *Left column*, *top*: photon spectra integrated over the whole polar angle 0 ≤ *ϑ* ≤ 20 mrad (blue curve), over the polar range 0 ≤ *ϑ* ≤ 10 mrad (green curve) and over the polar range 10 ≤ *ϑ* ≤ 20 mrad (red curve). *Left column, bottom*: angular distribution of the X-ray photons (integrated over the whole energy range). *Middle column*: photon angular distributions, taking into account only “low-energy” photons (0 ≤ *E*_*ph*_ ≤ 40 keV), *top*, or “high-energy” photons (40 ≤ *E*_*ph*_ ≤ 80 keV), *bottom*. *Right column*: Angular map of the average photon energy (*top*) and of the photon energy standard deviation (*bottom*). The angular maps span an azimuthal (*φ*, as shown in Fig. 1) range 0 ≤ *φ* ≤ 2*π* and a polar angle (*ϑ*) range 0 ≤ *ϑ* ≤ 20 mrad.

The X-ray source features a transverse size of around 2 *μ*m FWHM. The X-ray pulse duration, in the TS geometry considered here, is comparable to the electron bunch duration, and thus is in the sub-10 fs range^40,41^. Figure 2 shows, in the left column, the spectrum of the X-ray photon beam (top) and the photon angular distribution (bottom) as calculated using the TSST code. In the TS linear regime used here the spectrum extends up to the energy corresponding to the so-called fundamental harmonics, ${E}_{X}\simeq 4{\bar{\gamma }}^{2}{E}_{L}$, being $\bar{\gamma }$ the average *γ* factor of the electron bunch and *E*_*L*_ = *ħω*_*L*_ the photon energy of the scattering beam^33^. As it is usual for a TS source, there is an angular dependence of the energy spectrum; this can be observed looking at the three curves in the first (top left) plot, which shows the photon spectrum integrated over different polar angle intervals. In particular, higher energy photons are predominantly emitted at smaller polar angles (0 ≤ *ϑ* ≤ 10 mrad), while relatively low energy photons appear at angles *ϑ* > 10 mrad. The angular distribution is rather elongated along the *x* (*φ* = 0) direction; this corresponds to the polarization direction of the scattering laser beam. The anisotropy is mostly typical of lower energy photons, as it can be realized looking at the plots in the middle column, which show the angular distribution for “low” energy (0 ≤ *E*_*ph*_ ≤ 40 keV) and high (40 ≤ *E*_*ph*_ ≤ 80 keV) energy photons, respectively. The right column shows the average photon energy (top) and the energy standard deviation (bottom) as a function of the emission angles. For the sake of the application discussed in this paper, we choose our *μ*CT geometry (basically SAD and ADD) in order to only rely on photons emitted within *ϑ* ≤ 10 mrad. Over such a range, the average photon energy is between 35 and 50 keV, and the standard deviation is of about 15 keV. We carried out extensive simulations in order to highlight possible effects of this real spectral/angular photon distributions on the quality of the final image; we will discuss this issue below.

## Results

The ability of the system just described to reconstruct 4D images of the heart of the living animal was demonstrated by mean of analytical simulation of projection data with synthetic additive Poisson noise. The parameters of the idealized 4D numerical phantom are reported in the Supplementary Materials. The main parameters of the analytical simulation are reported in Table 2 (right column). In the Supplementary Material, additional simulations are reported to highlight the beneficial effect of ultrashort pulse duration on the proposed application. The peak myocardial wall velocity during systole was set to 45 mm/s in this simulation, based on micro-ultrasound measurements (see Supplementary Materials) and on previous studies on murine kinetics of cardiac contraction^42,43^. At this velocity, the wall displacement at systole is >400 *μ*m over an integration time of 10 ms, which is a typical X-ray pulse duration or detector integration time for conventional 4D *μ*CT instrumentation. In order to get near blur-free imaging, the wall displacement should not exceed $\sim 50\,\mu m$, i.e., half of the reconstruction voxel size which can be set to 100 *μ*m without compromising morpho-functional cardiac measurements in mice. Hence, the required temporal resolution is in the order of 1 ms or lower. However, due to constraints on experiment duration (number of pulses required) and radiation dosimetry, a more realistic requirement for robust 3D strain/strain rate analysis is to set the temporal binning around 2–3 ms per cardiac phase as already done with high-field MRI on rats^8^. Such requirements can be relaxed for the other phases of the cardiac cycle. In this work, we have set the temporal binning to 3 ms throughout the entire cardiac cycle.

### 4D cardiac imaging results with realistic cardiopulmonary motion

**Figure 3:**
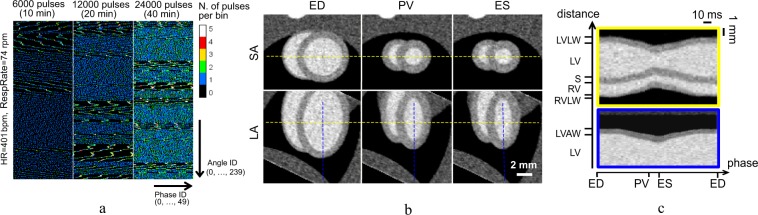
(**a**) 2D histograms showing the distribution of the X-ray pulses on the phase/angle diagram for the simulated acquisition of the dynamic phantom. Histograms are binned in 50 cardiac phase bins and 240 angular bins. Dark areas show missing data due to transitory coherence between the cardiac motion and the X-ray pulse sequence. (**b**) Reoriented short-axis and long-axis images of the mouse heart (only three of the 50 reconstructed phases are shown) obtained by iterative reconstruction from the sequence of 12000 pulses. (**c**) M-mode-like image of the reoriented heart, obtained along the dashed lines shown in (**b**) for all the 50 reconstructed time frames. For all images, the voxel size is 100 *μ*m and the temporal binning (frame duration) is 3 ms, calculated as the mean R-R interval divided by the number of reconstructed frames per cycle. ED: end-diastole; PV: peak myocardial velocity; ES: end-systole; SA: short axis; LA: long axis; LV: left ventricle; LVLW: left ventricle lateral wall; LVAW: left ventricle apical wall; RV: right ventricle; RVLW: right ventricle lateral wall; S: septum. FBP: filtered backprojection; SIRT: simultaneous iterative reconstruction technique.

A simulation was performed using the retrospectively gated acquisition strategy specifically foreseen for our 4D *μ*CT prototype. Due to the real X-ray pulse duration expected for the TS source, in the order of a few up to ~10 femtoseconds, we have neglected the finite pulse duration in this simulation (Δ*T*_*pulse*_ = 0 ms). We have generated a time series of 2D X-ray projections at constant repetition rate of 10 Hz of the 4D numerical phantom. Using real ECG and respiration waveforms previously acquired on mice and rats in our laboratory (see Supplementary Materials), each 2D projection was generated at a specific cardiac and respiratory phase; an average heart rate of 401 bpm and respiration rate of 74 respiratory acts per minute where used. A total number of pulses ranging from 6000 to 24000 over a full rotation of 360° was employed. As shown in Fig. 3a, these pulse sequences covered almost uniformly the whole cardiac cycle on most angular bins, allowing good flexibility in the choice of the subsequent temporal binning. In most cases, transitory coherence between the X-ray repetition rate and the cardiac motion was observed, randomly inducing missing data at specific ranges of projection angles and cardiac phases (Fig. 3a). Such missing data is a common source of streak artifacts in retrospectively gated acquisitions when analytical reconstruction (such as filtered backprojection) is performed. To reduce these artifacts, we employed the simultaneous iterative reconstruction technique (SIRT)^44^. Figure 3b shows the reconstructed heart obtained by SIRT at end-diastole (ED) and end-systole (ES), after 300 iterations from the acquisition sequence of 12000 pulses and reoriented in short-axis (SA) and long-axis (LA). With an average R-R time of ~150 ms, we have obtained a temporal sampling of 3 ms by rebinning the projection data in 50 temporal frames. The selected respiratory window was 10%–90% (relative to the peak inspiration time), so that only 9632 projections out of a total of 12000 were employed in the reconstruction. M-mode-like reconstructions of the cardiac wall along two perpendicular directions are shown in Fig. 3c.

**Figure 4:**
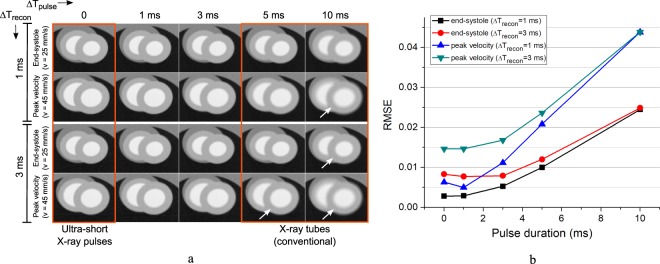
Noiseless simulation showing the motion blurring for different duration of the reconstruction window Δ*T*_*recon*_ and the X-ray pulse Δ*T*_*pulse*_, reconstructed at end-systole (*v* = 25 mm/s) and at time of peak myocardial velocity (*v* = 45 mm/s). (**a**) Reconstructed images in the transaxial plane (*xy*); the white arrows indicate settings leading to non negligible motion blurring. All images have been reconstructed by FBP with a voxel size of 100 *μ*m from 240 projections per cardiac phase, taken at 1.5° sampling interval. (**b**) RMSE analysis relative to a reconstruction of the same ROI otained with a fixed reconstruction window of negligible duration, Δ*T*_*recon*_ = 1 *μ*s (reference image not shown).

In order to highlight the advantage of using X-ray pulses much shorter than obtainable in conventional micro-CT instrumentation, we have also performed additional simulations without adding noise, by varying the duration of the X-ray pulse and of the reconstruction window. The simulations were performed on the end systolic phase (*v* = 25 mm/s) and at the phase of peak velocity (*v* = 45 mm/s) which is the most demanding phase in terms of spatial resolution when a 3D strain rate analysis is to be performed. The results are shown in Fig. 4. Even for this relatively coarse voxel size (100 micron), the RMSE error is nearly doubled when a pulse duration of 3 ms is used instead of ultra-short pulses, for a reconstruction time window of 1 ms. When using typical pulse duration obtainable in conventional micro-CT instrumentation (Δ*T*_*pulse*_ = 5–10 ms), the image quality degradation is evident even from a qualitative point of view, especially for the phase of peak velocity. No apparent differences can be found for pulse duration of 1 ms or below, due to the large (but realistic) voxel size used in this simulation.

### Dosimetry and image quality assessment using Monte Carlo simulations with TS spectrum and fluence

In order to assess the possible effects of the peculiar spectral and angular features of the TS X-ray beam on the quality of the final image for the kind of study proposed here, Monte Carlo simulations of the photon transport through a standard phantom and of the energy deposition on a conventional (phosphor screen based) X-ray detector were carried out, using the TS source photon distribution provided by the TSST code. These simulations also allowed a dosimetric study of the envisioned CT device to be performed. The phantom was made up by an 18 mm diameter water cylinder; at its center, a smaller cylinder, with diamter 5 mm, was supposed to be filled with an iodinated contrast agent with 3 mgI/mL. The source-to-phantom and the source-to-detector distances were set as in the analytical simulation (see Table 2, right column). Further details on these simulations are reported in the Methods Section and in the Supplementary Materials (examples of the synthetic images retrieved are also shown there).

**Figure 5:**
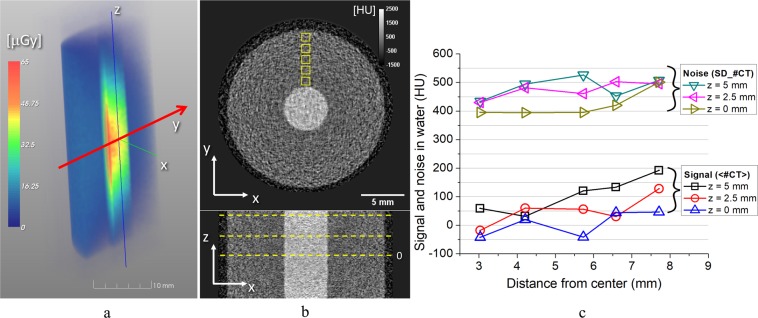
(**a**) Volume rendering of the 3D distribution of the X-ray dose delivered by a single laser shot to the water phantom with iodine insert (see text). For visualization purposes, a virtual cutting on the *yz* plane has been made on this rendering. The red arrow (*y* axis) shows the main direction of propagation of the X-ray beam for the selected laser shot. (**b**) Transverse (top) and coronal (bottom) slices of the tomographic reconstruction of the same phantom in (**a**). All images are reconstructed with a SIRT algorithm, 300 iterations, and with an isotropic voxel of 100 *μ*m. (**c**) Average reconstructed values (signal) and noise in water at several radial and axial positions. The radial positions of the ROI’s are depicted as yellow squares in (**b**), whereas the axial positions are shown as dashed lines in the coronal image (only *z* = 0 is marked).

Figure 5a shows a 3D density map of the dose deposition inside the phantom by a single laser shot (that is, according to the TSST simulation discussed above, considering ~10^8^ photons per shot). A cubic averaging volume with a side length of 0.5 mm was used to reconstruct a raster image from this single-shot dosimetric simulation. The average phantom dose delivered with a single laser shot was 11.7 *μ*Gy; the entrance surface dose was 11.3 *μ*Gy.

For each X-ray pulse, we have measured the energy deposition onto a 100 *μ*m thick Gadox (Gd_2_O_2_S) phosphor screen^45^, with pixel size and active area as in Table 2. We ran a total of 240 projections over 360° (i.e, similar to the average number of projections per cardiac phase employed in the simulations shown above in Fig. 3b,c)), with uniform angular sampling step of 1.5°. Each view was corrected with a flat field, which, in turn, was obtained by averaging 100 independent projections with no phantom in the field of view. A few projection images produced with the Monte Carlo code are reported in the Supplementary Material. A SIRT algorithm with 300 iteration was used to reconstruct the CT volume, with an isotropic voxel size of 100 *μ*m as already done in the previous subsection. Figure 5b shows transverse and axial slices of the tomographic reconstruction; an increasing trend of the average reconstructed value toward the phantom periphery is observed, primarily due to the beam hardening effect. Due to the relatively higher energy spread at the FOV periphery (see Fig. 2), this effect was more evident on the slice reconstructed at *z* = 5 mm, where the average CT number in water increased from 59 HU at *r* = 3 mm to 192 HU at *r* = 7.7 mm (denoting by $r=\sqrt{{x}^{2}+{y}^{2}}$ the radial distance from the slice center). An increasing trend of the image noise toward the FOV periphery is also observed. In the midplane (*z* = 0), the image noise in water increased from 395 HU at *r* = 3 mm to 501 HU at *r* = 7.7 mm. This is due to the decreasing average photon energy and photon fluence away from the central beam axis (see Fig. 2). For the same reasons, the average signal and noise of the iodine insert increased from 1189 ± 383 HU at the midplane to 1410 ± 478 HU at *z* = 5 mm. The average and standard deviation of contrast to noise ratio (CNR) of the iodine insert was 3.0 ± 0.3. This is fully compatible with a subsequent image post-processing and ventricle segmentation for morphometric analysis.

## Discussion and Perspectives

A compact imaging platform based on an all-optical laser-based Thomson scattering X-ray source has been conceived, designed and fully simulated by means of analytical and Monte Carlo simulations. To the best of the authors’ knowledge, no studies (numerical or experimental) have been conducted so far on the possible use of all-optical Thomson scattering ultrashort X-ray sources for the specific task of cardiac 4D imaging of the small rodent. As discussed above, none of the current standard imaging modalities devoted to dynamic morphofunctional quantification of small animal heart can achieve, at the same time, isotropic 3D spatial resolution in the order of 100 *μ*m and temporal resolution in the order of (or better than) 1 ms, as required for robust 3D cardiac strain and strain rate analysis in small animals. This gap is due to limitations of both current technology of compact X-ray sources (i.e., available in small-scale laboratories) and readout speed of high resolution X-ray detectors. Laser-based TS sources, as discussed in this paper, appear the most promising alternative to 3rd and 4th generation synchrotron sources, providing extremely short pulse duration, high spatial coeherence and relatively small footprint and cost^15,46^, and thus paving the way for the spread of X-ray sources with spatial and temporal features boasted so far only by large scale infrastructures.

We have demonstrated here that, using the proposed 4D *μ*CT prototype based on TS source, it is feasible to obtain motion blur free 3D images of the mouse heart at isotropic voxel size of 100 *μ*m with wall velocities up to 45 mm/s, which is generally higher than what practically found in mice and rats, especially under deep anesthesia^42^. An attractive feature of our TS-based 4D *μ*CT prototype is that, due to the extremely short duration of the X-ray source, no time overlap can occur between X-ray pulses at any phase of the cardiac cycle; this means that the reconstructed time bin duration *T*_*recon*_ can be set to an arbitrarily short duration without any overlap of adjacent phases, provided that a sufficient amount of projections per angle is acquired for each phase. Hence, the only practical limit to the temporal resolution is given by the radiation dose and the experiment duration. The temporal precision of R-peak identification on the ECG signal, along with the correlation between ECG and cardiac motion periodicity, could also play a role in the final temporal resolution of long gated acquisitions with the proposed system. These latter limitations are currently disregarded in conventional 4D *μ*CT, as they can only have an effect when sub-ms temporal resolution is pursued. The estimated radiation dose after 12000x-ray pulses generated during the simulated retrospectively gated acquisition was $\lesssim 150\,{\rm{mGy}}$. This value is comparable to or lower than doses reported on similar works using conventional sources^9,47–49^. Even though the focus of this paper was not on accurate dosimetry, the GEANT4 simulation toolkit has been already shown to be accurate in dosimetric calculations at low photon energy^50^. Employing as many dose-lowering setup parameters as possible is beneficial in the proposed setup to push the temporal resolution of the system close to its fundamental limit, which is related to the synchronization between each laser pulse and the actual phase in the R-R interval. The employment of SIRT reconstruction, besides its beneficial effect in the suppression of streak artifacts from irregular angular sampling, played also a beneficial role in the noise reduction and hence on the reduction of radiation dose. Besides this, the employment of a Gadox scintillator relatively thicker than standard for small animal imaging (100 *μ*m) and a mean photon energy just above the iodine K-edge (35–50 keV in the selected FOV position) were beneficial to keep the dose low enough to allow margins of improvement in the trade-off between the achievable spatial and temporal resolution. The increased scintillator thickness is not an issue for the target spatial resolution of our application, as Gadox thickness up to 120 micron still provide a detector resolution >6 lp/mm at 10% MTF^51^. The overall spatial resolution of the tomographic system can be estimated using analytical models^52^; in our geometry, it will be <60 micron FWHM which is fully compatible with cardiac morphometry in small animals.

Apart from dose consideration, the experiment duration is also a limiting factor to be carefully taken into account. The laser repetition rate chosen for our simulation (10 Hz) lead to an experiment duration of 20 minutes in order to get a 4D reconstruction at 3 ms of temporal binning with enough image contrast to perform 3D strain analysis in mice. Even though thermoregulatory devices are demonstrated to be effective to keep stable the physiological parameters of mice for experiment durations up to 30 min^53^, longer acquisition times might introduce artifacts due to both myocardial uptake and blood pool washout of the contrast agent^54^. Hence, the total experiment duration should never exceed 30–40 minutes in total. Scaling up the laser rep rate to 100 Hz is fully feasible^55^, even though the present technology of low-noise, high resolution X-ray detector is still restricted to slower frame rates. The temporal characteristics of the selected detectors should be carefully taken into account when planning for such a source-related improvement. Prospective double cardio-respiratory gating appears unfeasible with laser-based sources, at least at actual stage of development, due to the inability to trigger laser shots from external sources and with random rates. Even though the X-ray pulses with fixed rep-rate could be put in AND with a separated physiological trigger signal^56^, in our design we preferred to employ the retrospective gating approach for the sake of experiment shortening, easiness of implementation and flexibility in the post-acquisition spatio-temporal binning. The selected TS-based source implies a scanning geometry with the animal placed on a vertically oriented cradle; even though this is less than optimal in terms of physiological stability of the animal when compared with standard rotating-gantry geometries, vertical positioning of the animal was already employed successfully in several previous works at synchrotron facilities^57^, other laser-based X-ray sources^58^ and even in special designs of micro-CT’s based on conventional X-ray tubes^48^.

We recognize that a similar approach to cardiopulmonary imaging in mice may be implemented at existing beamlines of many synchrotron facilities. Nevertheless, it must be understood that the advantage of obtaining comparable results with compact sources (potentially available at most biomedical laboratories or hospitals) is extremely important in almost all preclinical experiments, requiring the proximity to a multidisciplinary environment with biology/veterinary staff and other imaging modalities *in situ*. As a matter of fact, the dynamic cardiac microtomography proposed in this work is likely to remain at the proof-of-principle stage unless more compact and affordable sources, such as the all-optical TS source considered here, become available.

The kind of apparatus described here is currently under development at the Intense Laser Irradiation Laboratory (ILIL) of the CNR in Pisa, Italy, in the framework of the Italian project “PREclinical Laser-based Ultrafast Diagnostics and thErapy (PRELUDE)”. It is worth stressing here that laser-driven electron accelerators have witnessed a tremendous improvement of the final beam quality, in terms of beam divergence/emittance, total charge energy spread, and so on, so that this kind of technology can be considered at the mature level to reach in short times the clinical quality. For instance, the EuPRAXIA project^59^, whose preparatory phase is currently funded by EU in view of the preparation of a Conceptual Design Report, is aimed at developing a plasma-based accelerator with industrial beam quality and user areas for real applications in different fields, including biology and medicine^60^.

Apart from synchrotron sources, the proposed TS source is the only one capable of providing, at the same time, (i) ultra-short X-ray pulses, with (ii) photon energy spectra tunable in the optimal range for small animal imaging, and (iii) with a source size under 10 *μ*m and thus capable of enabling phase contrast edge enhancing, all within a small-scale laboratory infrastructure. A thorough investigation of the possible beneficial effects and possibilities open up by the point (iii) is beyond the scope of the current paper and will be carried out in a future work.

## Methods

### Ethical statement

All the animal experiments from which data has been retrieved to generate the numerical mouse chest phantom were conducted in compliance to local approved protocols at CNR-IFC, under Italian Law 26/2014 and 2010/63/EU and approved by the Italian Ministry of Health.

### Analytical dynamic simulations

Analytical simulation have been performed with a custom developed ray-tracing algorithm, calculating the intersection lengths of rays and ellipsoids arbitrarily oriented in the 3D space. Simulation of finite duration X-ray pulses was done by averaging 10 equally spaced instantaneous (Δ*T* = 0) pulses in the desired time frame. Because analytical simulations were performed with the main goal of evaluating the effect of pulse duration and of the reconstruction artifacts due to angular sampling non uniformity, the finite source size and the real X-ray spectrum were not simulated. Statistical noise with Poisson probability density function was added to each analytically simulated 2D projection by considering the attenuated number of photons in each detector pixel, normalized to a number of unattenuated photons (detected after just air) of 5000. This number is realistic when taking into account the results of the TS simulated photon output at the center of the detector, at 2500 mm from the e^−^-laser interaction where X-rays are originated, and by considering a detector binning with an effective pixel size of 250 × 250 *μ*m^2^. The attenuation coefficient of air has been neglected. More details on the 4D mouse chest numerical phantom can be found in the Supplementary Materials.

### The TS source simulations

The detailed spectral and angular features of the TS source proposed for the present study were retrieved using the *Thomson Scattering Simulation Tools (TSST)* code^32^. The code evaluates the far-field distribution of the radiation emitted incoherently by the electron bunch. The spectral and angular distribution of the X-rays emitted by a single electron are computed using a semi-analytical approach based on the formulas reported in^32^. The exact analytical description of the radiation emitted within a single laser cycle is then coherently summed (in a numerical fashion) by taking into account the exact longitudinal pulse profile. A head-on (*backscattering TS*) collision geometry was considered in our case, with the electron bunch and scattering laser figures listed in Table 2. The photon distribution function was sampled using 200 energy bins in the range 1–100 keV, 80 polar angle (*ϑ*) bins in the range 0 ≤ *ϑ* ≤ 20 mrad and 64 azimuthal (*φ*) bins in the range 0 ≤ *φ* ≤ 2*π*.

### Monte Carlo code for dosimetry and image quality assessment

A Monte Carlo code was developed in order to both model the imaging capability of the TS source and to perform a dosimetric study. The code was based on the Geant4 toolkit^61–63^. The low-energy physics models based on PENELOPE^64,65^ were used for the simulation of the electromagnetic interactions of X-rays. The primary photons were generated according to the energy and angular distribution provided by TSST code, using a rejection sampling method.

## Supplementary information

Supplementary Info

## Data Availability

The datasets generated during and/or analysed during the current study are available from the corresponding authors on reasonable request.
