# Supplementary material for: Numerical simulation of novel concept 4D cardiac microtomography for small rodents based on all-optical Thomson scattering X-ray sources
Source: Sci Rep. 2019 Jun 11;9:8439. doi: 10.1038/s41598-019-44779-y (PMC6560041; doi:10.1038/s41598-019-44779-y)
Supplement: Supplementary file 1 — Supplementary Info [file 41598_2019_44779_MOESM1_ESM.pdf]

Supplementary material for ”Numerical simulation of novel concept 4D cardiac microtomography for small rodents based on all-optical Thomson scattering X-ray sources”

Daniele Panetta, Luca Labate, Lucia Billeci, Nicole Di Lascio, Giuseppina Esposito, Francesco Faita, Giovanni Mettivier, Daniele Palla, Luciano Pandola, Pietro Pisciotta, Giorgio Russo, Antonio Sarno, Paolo Tomassini, Piero A. Salvadori, Leonida A. Gizzi, Paolo Russo

1 Numerical 4D mouse chest phantom and forward projector

The digital phantom used for the analytical simulations of the 4DμCT scanner is based on a superimposition of ellipsoids, for whose the computation of ray-object intersection lengths can be computed efficiently by mean of standard linear algebra routines. Even though other numerical phantom for mouse and rats have been developed in recent years [1], we developed here a new phantom with the precise intent of having high flexibility in the choice of heart kinetics parameters, by allowing an arbitrary number of harmonics and relative amplitude and phases to a main sinusoidal oscillatory motion of all the geometrical parameters describing the ellipsoids. In order to reproduce by ellipsoids a realistic mouse chest anatomy, we have used previously acquired 4DμCT datasets of living mice ($N = 3$) and rats ($N = 4$), reconstructed with temporal resolutions of 10-20 ms per frame, with up to 20 frames per cardiac cycle. The selected animals were chosen by our veterinary staff, among those available in our database, as the most representative healthy subjects when cardiopulmonary dynamics is concerned. An iodinated contrast agents was continuously perfused during the imaging study, as already described in Ref [2]. The IRIS micro-CT scanner (Inviscan SAS, Strasbourg, France) was used in all the CT experiments. All the animal experiments were conducted in compliance to local approved protocols at CNR-IFC, under Italian Law 26/2014 and 2010/63/EU.

The list of simulated organs and their geometrical parameters are reported in Table 1. We have put emphasis on the myocardial wall motion and respiratory motion, while the other organs where just reproduced to roughly mimick the whole-body attenuation of the X-ray beam as well as the bone-induced beam hardening artifacts. The numerical densities selected for our simulated experiments (see main text) were chosen in such a way that the myocardium/blood contrast was comparable to what obtained in-vivo by mean of continuous infusion of iodinated contrast agents. Even though atrial geometry and valves were not reproduced, we believe that the medial to apical segments of the ventricles are good approximation of the real cardiac anatomy. Other endocardial irregularities (such as papillary muscles) are planned to be added in the next future in order to include torsional motion for the simulation of circumferential strain. At this stage, only radial and longitudinal strain can be simulated.

Radiographic projections are obtained in our code by analytically computing ray-ellipsoid intersections, with an arbitrary size and number of detector pixels and with an arbitrary temporal resolution. When more than one ellipsoid is found along the ray trajectory, the priority for the computation of the line integral is given to the object with higher Id (see Table 1). Each parameter of each ellipsoid is assigned with a set of

Id	Organ	a_x, a_y, a_z (mm)	c_x, c_y, c_z (mm)	α_x, α_y, α_z (deg)	Density (a.u.)	Dynamic
1	Trunk	8.8, 9.2, ∞	0.5, -0.5, 0	0, 0, 0	1	
2	Lungs	6.2, 5.2, 10	0.5, -1.0, 2	-7, 0, 0	0.5	•
3	Atria	3.7, 3.5, 5.0	1.0, -3.8, -5.5	7, -30, 0	1	•
4	Epicardium (RV)	2.4, 2.4, 3.6	0.1, -3.8, -1.5	8, 35, 0	1.2	•
5	Endocardium (RV)	2.0, 2.0, 3.2	0.1, -3.8, -1.5	8, 35, 0	1.5	•
6	Epicardium (LV)	2.4, 2.4, 3.4	0.1, -3.8, -1.5	8, 35, 0	1.2	•
7	Endocardium (LV)	1.6, 1.6, 2.8	1.9, -3.4, -1.5	8, 35, 0	1.5	•
8	Liver	8, 8, 5	0.5, -1.5, 8.2	0, 0, 0	1	•
9	Aorta	1.0, 0.75, ∞	1.0, 2.2, 0	-7, 0, 0	1	
10	Spine (bone)	1.2, 1.2, ∞	0, 4.1, 0	-7, 0, 0	1.8	
11	Spine (marrow)	0.8, 0.8, ∞	0, 4.1, 0	-7, 0, 0	1.3	
12	Sternum	0.9, 0.6, ∞	0.5, -8.5, 0	0, 0, 0	1.7	
13	Trachea	0.6, 0.6, 5.5	0.5, -0.5, -8.5	-8, 0, 0	0.01	•
14	Left bronchus	0.4, 0.4, 2.8	2.0, 0.6, -8.0	-8, 40, 0	0.01	•
15	Right bronchus	0.4, 0.4, 2.8	-1.0, 0.6, -8.0	-8, -40, 0	0.01	•

Table 1: Geometrical parameters of the 4D numerical phantom, where $a_{x,y,z}$ are the ellipsoid semi-axis length, $c_{x,y,z}$ are the center coordinates, and $\alpha_{x,y,z}$ are the rotation angles around the main axis. An infinity sign indicates a size much greater than the other two dimension, which is used for some organ to simulate elliptic cylinders.

Fourier coefficients, phases and principal period in order to allow an arbitrary periodic motion. The actual time, expressed as a fraction of one period, can be set independently for each ellipsoid. When a finite X-ray pulse duration (or detector integration time) shall be simulated, the sum of the density-length products of all ellipsoids (i.e., the line integral) is computed several times (10 times in our experiments, as explained in the Methods section of the main text); then, the final value assigned to a detector pixel is computed as the average of all the line integrals computed at each time point. Statistical noise is added on the final image by first converting the line integral map of each view to the corresponding number of detected photons on each pixel. More precisely, for a given value $g_{i,j}$ of the line integral at the pixel location i, j, the value $N_{i,j,}$ of the detected photons at the same pixel location is computed as:

$$N_{i,j} = N_0 e^{-g_{i,j}}, \tag{1}$$

where N_0 is an arbitrary normalization constant chosen as the expected average number of unattenuated photons per pixel detected at the center of the detector. Once the noiseless direct radiography $N_{i,j}$ is computed, the noisy radiography $\hat{N}$ is calculated as $\hat{N}_{i,j} = N_{i,j} + n_{i,j}$, where n is a random number from a Poisson distribution with mean value $\bar{n} = N$. Finally, the noisy projection $\hat{g}$ is simply given by

$$\hat{g}_{i,j} = -\ln \frac{\hat{N}_{i,j}}{N_0}. \tag{2}$$

As explained in the Methods section of the main text, the value of N_0 in our analytical simulations was selected in order to match with the expected photon output of the Thomson scattering source. Hence, for the selected source-detector distances (2500 mm) and detector bin size (250^2 μm^2), we have set $N_0 = 5000$.

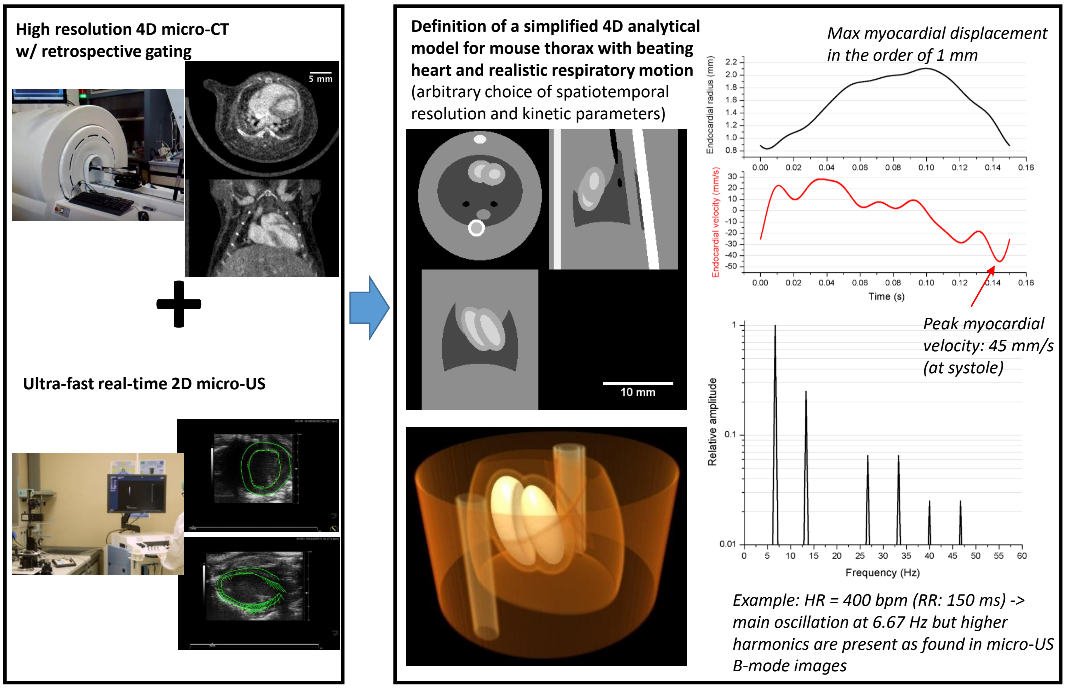

Figure 1: Schematic workflow used to build the 4D mouse chest. Micro-CT and micro-US mouse studies were used to obtain realistic anatomy and kinetic data of the living mouse chest.

2 Generation of realistic cardiopulmonary motion from micro-US data and ECG-derived R-R sequences

In order to derive a realistic intra-cycle cardiac motion for our 4D phantom, we have analyzed B-mode microultrasound images of living mice previously acquired using state-of-art small animal high-frequency ultrasound (Vevo 2100, Fujifilm Visualsonics Inc, Toronto, Canada). All the animal experiments were conducted in compliance to local approved protocols at CNR-IFC, under Italian Law 26/2014 and 2010/63/EU. Short-axis and long-axis cardiac images were acquired in real-time with a temporal resolution of 3.2 ms and 5 ms per frame, respectively, and with a spatial resolution of 30 μm. Images were subsequently analyzed using specific wall tracking software (VevoStrain Analysis, Fujifilm Visualsonics Inc, Toronto, Canada) obtaining 2D strain, strain rate, dispalcemente and velocity values for 48 and 49 points, in short-axis and long-axis images, respectively. Afterwards, frequency analysis of the displacement vector on each point was performed by fast Fourier transform (FFT) of the kinetic data.

Global modes of periodic ventricular motion was then obtained by averaging the FFT data of all analyzed tracked points, as shown in the right side of Figure 1. The phase of each frequency component was manually adjusted until a realistic myocardial displacement was obtained (see Figure 1, top right box). Even though 7 main harmonics have been added to our phantom based on the micro-US results, an arbitrary number of harmonics can be used in our code. Figure 2 shows the main differences in terms of kinetic parameters

between systole and diastole, obtained for our simulations as described above. At diastole, the myocardial wall velocity is always less than 1 cm/s, which means that the overall displacement is less than 100 μm over an integration time of 10 ms. Hence, this phase is generally well reconstructed even with conventional state-of-art micro-CT instrumentation with cardiac gating capability. At systole, the maximum myocardial velocity during contraction with the current phantom settings is 45 mm/s (=45 μm/ms) at time $t = 0.96\ T$, where T is the heart cycle period chosen as $T = 150$ ms in this simulation. These values are compatible to what observed in micro-US studies and with data found in the literature[3]. This means that, at systole, a wall displacement greater than 400 μm is obtained over an integration time of 10 ms.

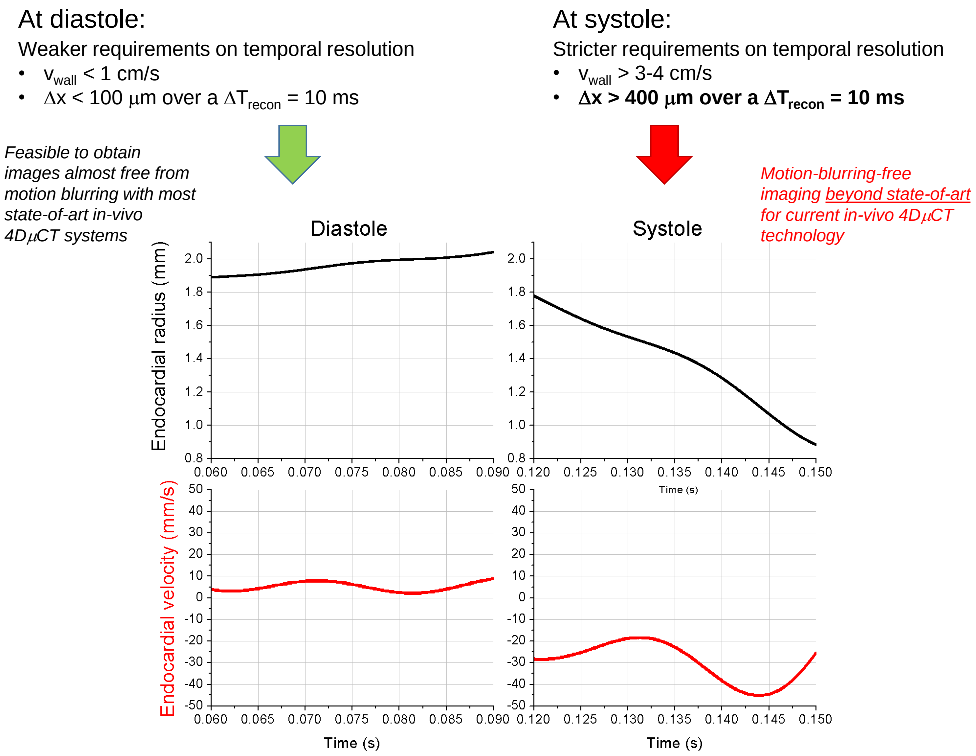

Figure 2: Differences in myocardial wall kinetics of the simulated phantom between diastole and systole.

Besides intra-cycle motion, we have also simulated realistic inter-cycle variability of cardiopulmonary motion by mean of previously acquired ECG and free-breathing motion signals, from which R-R sequences and respiration interval sequences have been obtained [4]. The template sequences are shown in Figure 3. Starting from these sequences, arbitrary rates for cardiac and respiratory motion of our 4D phantom are obtained by simple linear scaling of the time intervals of the template signals. Even though the cardiac and respiratory sequences are not physically correlated (i.e., they are coming from different animal studies) we believe that the obtained model of cardiorespiratory motion is realistic enough to allow numerical simulation of long gated imaging studies.

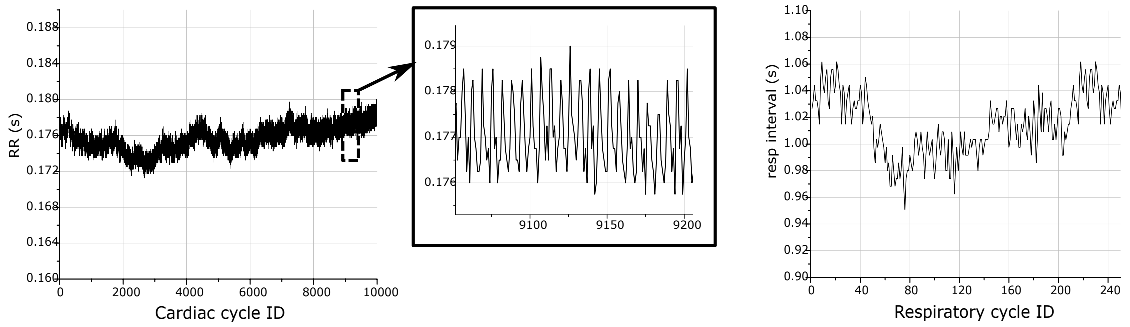

Figure 3: Typical sequences of R-R intervals (left box) and respiratory intervals (right box), as derived from physiological measurements on rat experiments at CNR-IFC. The inset shows a zoom of the long R-R signal, where low-frequency variability due to respiratory sinus arrhythmia (RSA) is visible.

3 Rebinning scheme for cardiac and respiratory retrospective gating

In the proposed TS-based prototype of 4DμCT, the physiological signals and the radiographic projections are taken simultaneously using a common time reference. This reference is provided by the laser timing electronics and/or an optical pulse. For instance, typical ultrashort femtosecond laser systems can provide a wealth of signals synchronized with the pump laser systems (that is, at times from a few up to a few tens of nanoseconds before the actual main laser pulse, with jitters of the order of a few picoseconds). Alternatively, an optical pulse can be picked up from the laser amplification chain, using a mirror leakage, and sent to a fast photodiode; timing jitter can be reduced to a few femtosecond range in this case. The animal cradle is continuously rotated in such a way that a full rotation (2π) is obtained after a fixed number of X-pulses coming from the TS source. Unlike prospectively gated acquisitions, requiring real-time ECG processing and the ability to trigger an X-ray pulse based on a external digital signal, our proposed prototype only allows retrospective gating based on the off-line analysis of the physiological signals and rebinning of the projection data. Both approaches are conceptually visualized in Figure 4. Due to the extremely short duration of the X-ray pulses, no time overlap can occur between X-ray pulses in any phase of the cardiac cycle; this means that the reconstructed time bin duration T_{recon} can be set to an arbitrarily short duration without any overlap of adjacent phases, provided that a sufficient amount of projection per angle is acquired for each phase. Hence, as explained in the main text, the only practical limits to the temporal resolution is given by the radiation dose and the experiment duration.

In our simulation, we have decided to use a uniform temporal binning of the event in the cardiac cycle, even though this does not prevent to refine adaptively the time bin duration depending on the actual wall velocity at each phase. As explained in the main text, the reorganization of projected images in the angle/phase diagram from an acquisition scheme as the one proposed here leads to angular sampling nonuniformity, which must be addressed by mean of specific iterative reconstruction algorithms such as the SIRT [5, 6]. The right box of Figure 4 shows how the angle/phase diagram is populated in practice. Each vertical strip of this diagram should contain enough data to reconstruct a full 3D tomographic image. Projections taken outside of a specified respiratory window may be rejected to avoid motion blurring due to global heart motion during the respiratory act.

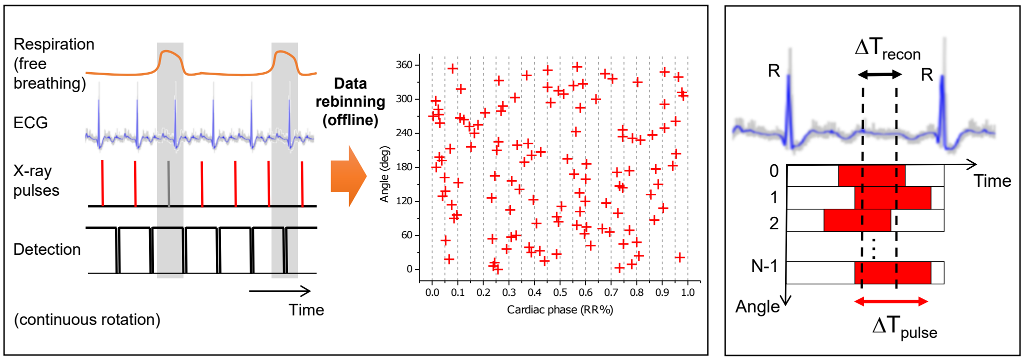

Figure 4: Prospective vs. retrospective gating strategies in cardiac micro-CT. In our simulations, the real data acquisition strategy planned for the Thomson scattering 4DμCT based on retrospective gating is shown on the left. Projections outside a defined respiratory window (grayed zones in the physiological signal plot) may be rejected to avoid residual motion blurring due to global heart motion during the respiratory act. Prospective gating, as shown on the right, was only used to the purpose of quantifying the motion-derived image blurring caused by the finite duration of the X-ray pulse in conventional sources.

4 Analytical vs iterative reconstruction

In order highlight the advantage of using the SIRT reconstruction instead of standard Filtered Backprojection (FBP) in our proposed TS-based 4DμCT system, we have performed the reconstructions shown in Figure 5b of the main text using both algorithms. The results are shonw in Figure 5. As expected, SIRT provided a better image uniformity in presence of the strongly irregular angular sampling due to the restrospective gating strategy in use.

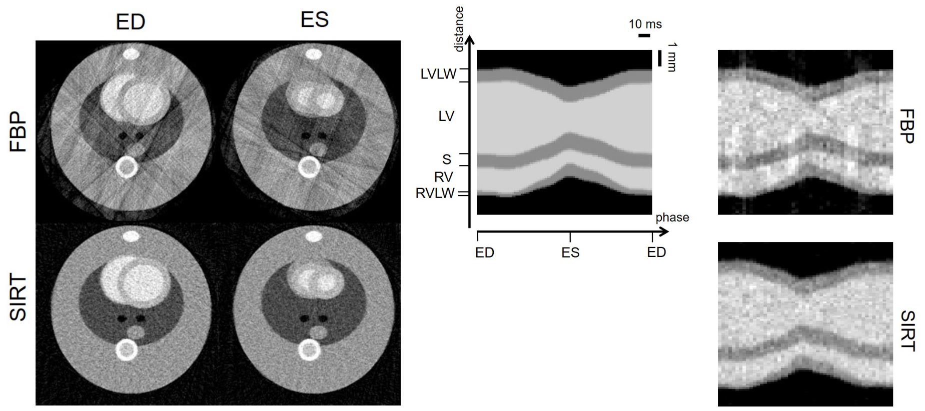

(a) (b)

Figure 5: (a) Comparison of FBP vs SIRT reconstructions of the analytical synamic simulations shown in Figure 5 of the main text. In (b), the M-mode-like images obtained with both algorithms are shown (right column), in comparison with the ground truth (top-left). ED: end-diastole; ES: end-systole; LV: left ventricle; LVLW: left ventricular lateral wall; S: septum; RV: right ventricle; RVLW: right ventricular lateral wall.

5 Monte Carlo simulations using the actual TS photon distribution

A custom Monte Carlo code was developed in order to both *a)* assess the imaging capabilities of the envisioned TS source and *b)* characterize the 4DμCT device proposed here from a dosimetric point of view. These two points are of a crucial importance in this case, in light of the peculiar spectral and angular distribution of the photons emitted by a TS source.

The code was based on the Monte Carlo Geant4 toolkit [7–9]. The Monte Carlo simulation accounts for the production and the propagation of secondary electrons, positrons and photons. The set of low-energy models based on PENELOPE[10, 11] were used for the accurate description of the electromagnetic interactions. The primary particles (photons) were generated according to the distribution function $\mathrm{PDF}(E, \vartheta, \varphi)$ provided by the TSST code (see text); to this purpose, a trilinear interpolation of the PDF and a rejection method were used [12].

An $18\,\mathrm{mm}$ diameter cylindrical water phantom was used, placed at a $1000\,\mathrm{mm}$ distance from the source. An inner cylindrical region of this phantom, with diameter $5\,\mathrm{mm}$ and axis overlapping to the axis of the bigger cylinder, was filled with an iodinated contrast agent with $3\,\mathrm{mg\,I/mL}$.

The image formation process was reconstructed by retaining the energy deposited on an ideal, $100\,\mu\mathrm{m}$ Gadox scintillator, with virtual pixels of $250 \times 250\,\mu\mathrm{m}^2$. The Gadox scintillator was considered to be $50 \times 25\,\mathrm{mm}^2$ large (the bigger size is orthogonal to the phantom cylinder axis in the image plane), and was placed at $1500\,\mathrm{mm}$ from the phantom.

Each image (i.e., simulation run) was obtained considering 10^8 primary photons, which corresponds to the photon flux (within the sampled region in the space E, ϑ, φ) per laser shot provided by the TSST code.

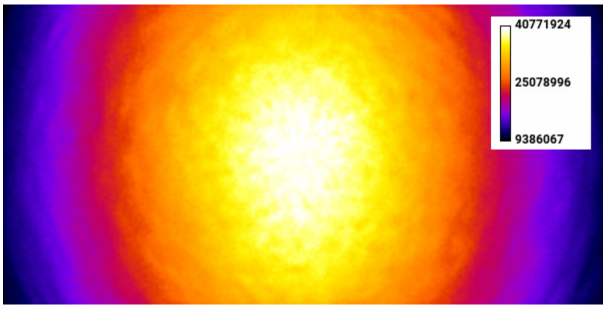

Figure 6: Energy deposited in each "pixel" (with size $250 \times 250\,\mu\mathrm{m}^2$) of the Gadox scintillator (in eV) in each laser shot, as simulated using our Geant4-based Monte Carlo code.

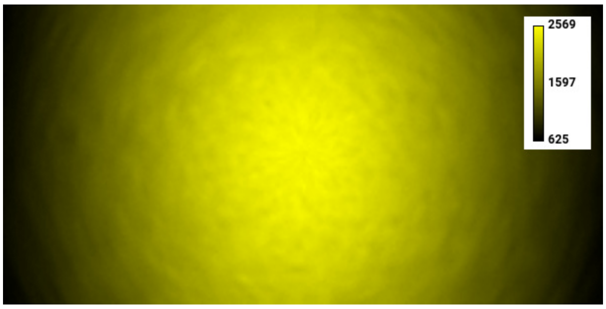

Figure 7: Number of photons impinging on each "pixel" (with size $250 \times 250\,\mu\mathrm{m}^2$) of the Gadox scintillator in each laser shot, as simulated using our Geant4-based Monte Carlo code.

The flat field was retrieved by running simulations without the presence of the phantom. Figures 6 and 7 show maps of the energy deposited and of the number of photons impinging on each Gadox pixels, respectively.

Figures 8 and 9 show the obtained images in the presence of the phantom, with and without a flat correction. Finally, Figure 10 shows a horizontal lineout of figure 9. This kind of image was finally used for SIRT reconstruction, as reported in the main text.

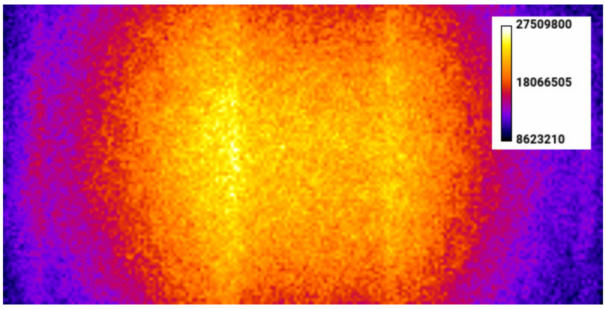

Figure 8: Raw (i.e., not flat corrected) image of the phantom described in the text as simulated using our Geant4-based Monte Carlo code. The gray scale represents the total energy (in eV) deposited on each scintillator "pixel" (with size $250 \times 250\,\mu\mathrm{m}^2$) per laser shot.

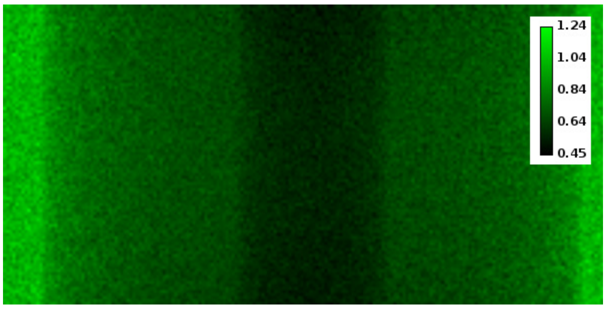

Figure 9: Flat-corrected image of the phantom described in the text as simulated using our Geant4-based Monte Carlo code.

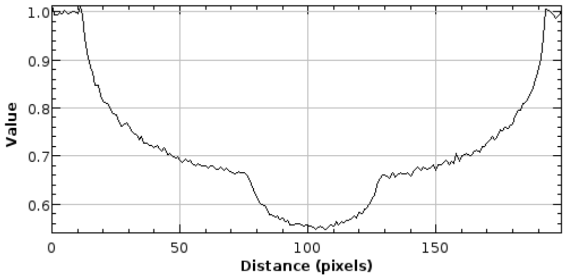

Figure 10: Horizontal lineout (averaged over the whole vertical extent) of Figure 9.

6 PIC simulations of the laser-driven electron acceleration stage

In this Section, a report of the results of the Particle-In-Cell (PIC) simulations of a possible LWFA regime providing the electron bunches driving the X-ray source are given. As it is clear from the discussion in the main text, we don't require, for the application proposed in this paper, a narrow spectrum of the X-ray beam, so that electron bunches with up to $15-20\%$ energy spread can be considered in this case. The PIC simulation was carried out using the quasi-3D code FBPIC [13]. A $25\,\mathrm{fs}$ duration, $75\,\mathrm{TW}$ laser pulse was supposed to be focused onto a gas-jet target down to a waist size of $25\,\mu\mathrm{m}$. The gas was supposed to be a mixture of He and 1.5% N_2, with a maximum electron density $n_e = 9 \times 10^{18}\,\mathrm{cm}^{-3}$. The plasma density longitudinal profile consisted, as it is quite standard when using supersonic gas-jets, of a linear up-ramp of length $150\,\mu\mathrm{m}$, a $600\,\mu\mathrm{m}$ long plateau (with density n_e) and a final linear down-ramp $150\,\mu\mathrm{m}$ long.

In order to get a moderately narrow spectrum, we relied on the so-called ionization-injection process [14]. The a_0 parameter (resulting from the above values) $a_0 \simeq 1.6$ was thus chosen so as to get newborn electrons extracted by field ionization $\mathrm{N}^{5+} \rightarrow \mathrm{N}^{6+}$ and trapped into the plasma wave. In Figure 11, a snapshot of the longitudinal electric field at the time at which the pulse has moved by about $200\,\mu\mathrm{m}$ in the plateau is given (along with a lineout on the axis). The longitudinal phase space of the extracted and accelerating particles, along with a lineout of the accelerating (longitudinal) and laser pulse (transverse) electric fields, is shown in Figure 12 at the same timestep. Finally, the electron energy spectrum just after the density downramp, where the interaction with the scattering laser pulse is supposed to take place, is shown in Figure 13, featuring a mean energy $E \simeq 50\,\mathrm{MeV}$ with an energy spread $\lesssim 20\%$, and a total bunch charge in excess of $100\,\mathrm{pC}$.

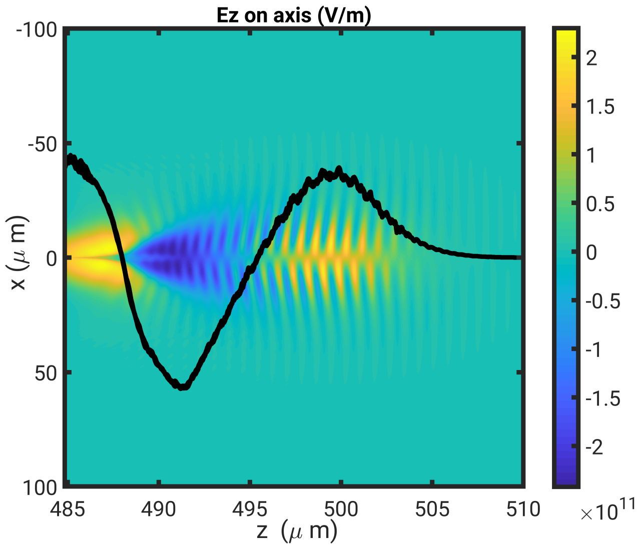

Figure 11: Snapshot of the longitudinal electric field at the time at which the laser pulse has moved by about $200\,\mu\mathrm{m}$ in the density plateau region, as provided by the PIC simulations. A lineout taken on the axis is also shown.

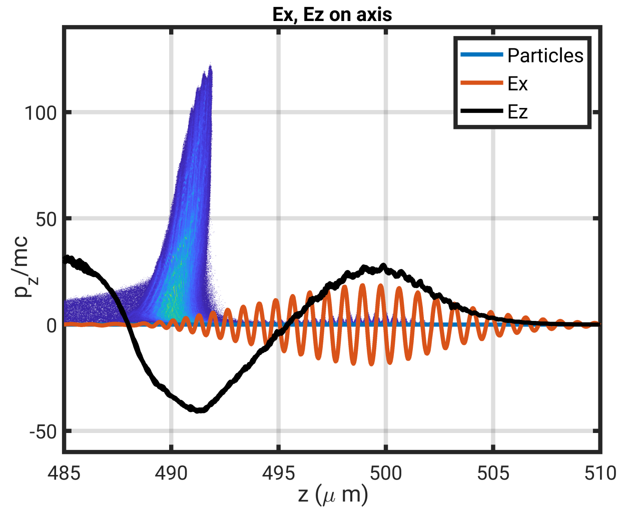

Figure 12: Longitudinal phase space of the accelerating particles taken at the same timestep of the previous Figure. Also shown are the lineouts of the accelerating (E_z) and the laser pulse (E_x) electric fields.

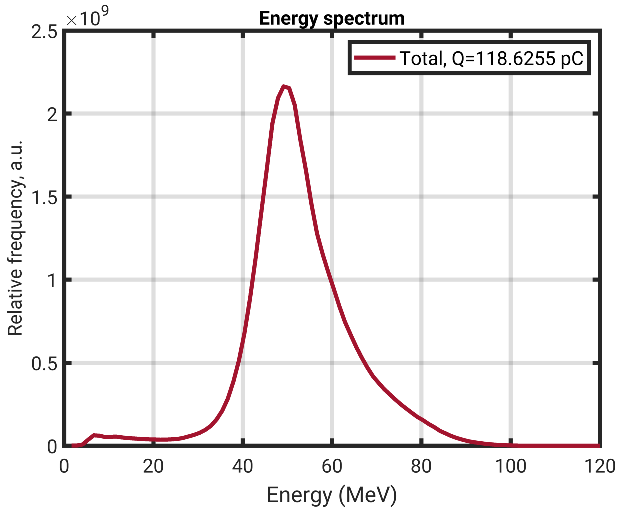

Figure 13: Energy spectrum of the accelerated electrons taken at the exit of the downramp. The retrieved total bunch charge is shown in the inset.

References

[1] W. P. Segars, B. M. W. Tsui, E. C. Frey, G. A. Johnson, and S. S. Berr, "Development of a 4-D digital mouse phantom for molecular imaging research," *Molecular Imaging & Biology*, vol. 6, pp. 149–159, May 2004.

[2] D. Panetta, N. Belcari, Silvia Burchielli, G. Pelosi, M. Tripodi, M. R. De Simone, P. Iozzo, A. Del Guerra, and P. A. Salvadori, "Temporal weighting and angular rebinning for artifact-free single-rotation restrospectively gated 4d cardiac micro-CT," in *Proceedings of the World Molecular Imaging Congress 2015, Honolulu, Hawaii, September 2-5, 2015*, vol. 18 of *Molecular Imaging and Biology*, pp. S356–S357, Springer International Publishing, July 2016.

[3] I. A. Sebag, M. D. Handschumacher, F. Ichinose, J. G. Morgan, R. Hataishi, A. C. T. Rodrigues, J. L. Guerrero, W. Steudel, M. J. Raher, E. F. Halpern, G. Derumeaux, K. D. Bloch, M. H. Picard, and M. Scherrer-Crosbie, "Quantitative Assessment of Regional Myocardial Function in Mice by Tissue Doppler Imaging: Comparison With Hemodynamics and Sonomicrometry," *Circulation*, vol. 111, pp. 2611–2616, May 2005.

[4] C. Del Seppia, D. Lapi, S. Ghione, G. Federighi, L. Sabatino, E. Fommei, A. Colantuoni, and R. Scuri, "Evidence in hypertensive rats of hypotensive effect after mandibular extension," *Physiological Reports*, vol. 6, no. 23, p. e13911, 2018.

[5] A. C. Kak and M. Slaney, *Principles of Computerized Tomographic Imaging*. IEEE Press, 1998. available online at http://www.slaney.org/pct/pct-toc.html.

[6] W. v. Aarle, W. J. Palenstijn, J. Cant, E. Janssens, F. Bleichrodt, A. Dabravolski, J. D. Beenhouwer, K. J. Batenburg, and J. Sijbers, "Fast and flexible X-ray tomography using the ASTRA toolbox," *Opt. Express, OE*, vol. 24, pp. 25129–25147, Oct. 2016.

[7] S. Agostinelli, J. Allison, K. Amako, J. Apostolakis, H. Araujo, P. Arce, M. Asai, D. Axen, S. Banerjee, G. Barrand, F. Behner, L. Bellagamba, J. Boudreau, L. Broglia, A. Brunengo, H. Burkhardt, S. Chauvie, J. Chuma, R. Chytracek, G. Cooperman, G. Cosmo, P. Degtyarenko, A. Dell'Acqua, G. Depaola, D. Dietrich, R. Enami, A. Feliciello, C. Ferguson, H. Fesefeldt, G. Folger, F. Foppiano, A. Forti, S. Garelli, S. Giani, R. Giannitrapani, D. Gibin, J. J. Gómez Cadenas, I. González, G. Gracia Abril, G. Greeniaus, W. Greiner, V. Grichine, A. Grossheim, S. Guatelli, P. Gumplinger, R. Hamatsu, K. Hashimoto, H. Hasui, A. Heikkinen, A. Howard, V. Ivanchenko, A. Johnson, F. W. Jones, J. Kallenbach, N. Kanaya, M. Kawabata, Y. Kawabata, M. Kawaguti, S. Kelner, P. Kent, A. Kimura, T. Kodama, R. Kokoulin, M. Kossov, H. Kurashige, E. Lamanna, T. Lampén, V. Lara, V. Lefebure, F. Lei, M. Liendl, W. Lockman, F. Longo, S. Magni, M. Maire, E. Medernach, K. Minamimoto, P. Mora de Freitas, Y. Morita, K. Murakami, M. Nagamatu, R. Nartallo, P. Nieminen, T. Nishimura, K. Ohtsubo, M. Okamura, S. O'Neale, Y. Oohata, K. Paech, J. Perl, A. Pfeiffer, M. G. Pia, F. Ranjard, A. Rybin, S. Sadilov, E. Di Salvo, G. Santin, T. Sasaki, N. Savvas, Y. Sawada, S. Scherer, S. Sei, V. Sirotenko, D. Smith, N. Starkov, H. Stoecker, J. Sulkimo, M. Takahata, S. Tanaka, E. Tcherniaev, E. Safai Tehrani, M. Tropeano, P. Truscott, H. Uno, L. Urban, P. Urban, M. Verderi, A. Walkden, W. Wander, H. Weber, J. P. Wellisch, T. Wenaus, D. C. Williams, D. Wright, T. Yamada, H. Yoshida, and D. Zschiesche, "Geant4—a simulation toolkit," *Nuclear Instruments and Methods in Physics Research Section A: Accelerators, Spectrometers, Detectors and Associated Equipment*, vol. 506, pp. 250–303, July 2003.

[8] J. Allison, K. Amako, J. Apostolakis, H. Araujo, P. A. Dubois, M. Asai, G. Barrand, R. Capra, S. Chauvie, R. Chytracek, *et al.*, "Geant4 developments and applications," *IEEE Transactions on nuclear science*, vol. 53, no. 1, pp. 270–278, 2006.

[9] J. Allison *et al.*, "Recent developments in Geant4," *Nuclear Instruments and Methods in Physics Research Section A: Accelerators, Spectrometers, Detectors and Associated Equipment*, vol. 835, pp. 186–225, 2016.

[10] J. Baró, J. Sempau, J. M. Fernández-Varea, and F. Salvat, "PENELOPE: An algorithm for Monte Carlo simulation of the penetration and energy loss of electrons and positrons in matter," *Nuclear Instruments and Methods in Physics Research Section B: Beam Interactions with Materials and Atoms*, vol. 100, pp. 31–46, May 1995.

[11] V. Ivanchenko, J. Apostolakis, and A. Bagulya, "Recent improvements in Geant4 electromagnetic physics models and interfaces," *Progress in Nuclear Science and Technology*, pp. 898–903, 2011.

[12] W. H. Press, S. A. Teukolsky, W. T. Vetterling, and B. P. Flannery, *Numerical Recipes in C*. Cambridge, USA: Cambridge University Press, second ed., 1992.

[13] R. Lehe, M. Kirchen, I. A. Andriyash, B. B. Godfrey, and J.-L. Vay, "A spectral, quasi-cylindrical and dispersion-free Particle-In-Cell algorithm," *Computer Physics Communications*, vol. 203, pp. 66–82, 2016.

[14] A. Pak, K. A. Marsh, S. F. Martins, W. Lu, W. B. Mori, and C. Joshi, "Injection and Trapping of Tunnel-Ionized Electrons into Laser-Produced Wakes," *Phys. Rev. Lett.*, vol. 104, p. 025003, Jan 2010.
